# Microplastics in *Cronius ruber*: Links to Wastewater Discharges

**DOI:** 10.3390/ani15101420

**Published:** 2025-05-14

**Authors:** Sofía Huelbes, May Gómez, Ico Martínez, Raül Triay-Portella, Miguel González-Pleiter, Alicia Herrera

**Affiliations:** 1 Marine Ecophysiology Group (EOMAR), Instituto Universitario de Investigación en Acuicultura Sostenible y Ecosistemas Marinos (ECOAQUA), Universidad de Las Palmas de Gran Canaria, 35017 Canary Islands, Spain; may.gomez@ulpgc.es (M.G.); ico.martinez@ulpgc.es (I.M.); alicia.herrera@ulpgc.es (A.H.); 2 Biodiversity and Conservation Group (BIOCON), Instituto Universitario de Investigación en Acuicultura Sostenible y Ecosistemas Marinos (ECOAQUA), Universidad de Las Palmas de Gran Canaria, 35017 Canary Islands, Spain; raul.triay@ulpgc.es; 3 Department of Biology, Faculty of Science, Universidad Autónoma de Madrid, 28049 Madrid, Spain; mig.gonzalez@uam.es

**Keywords:** microplastics, *Cronius ruber*, Canary Islands, wastewater discharges, textile fibers

## Abstract

Microplastic pollution in the ocean is a growing problem. It affects the entire ecosystem and, therefore, the species that inhabit it. Plastics can be filtered or ingested by organisms, entering and negatively affecting individuals. Among the populations affected are crustaceans. In previous studies, fibers have been found mainly in the stomach contents of these animals, although other types, such as pellets, have also been found. This study examines the presence of microplastics in *Cronius ruber*, an invasive crab species in the Canary Islands, and investigates their potential links to nearby wastewater discharges. A total of 63 crabs were sampled from four beaches in Gran Canaria in 2021, and their stomach contents were analyzed through alkaline digestion, filtration, and micro-Fourier transform infrared spectroscopy (micro-FTIR). Microplastics were detected in 52% of individuals; the particles averaged 0.7 ± 0.5 mm in length, with an average of 1.73 ± 1.02 particles per crab. Fibers constituted 89% of the microplastics, with blue and black being the predominant colors. Rayon, commonly used in textiles, was the most frequently identified polymer (52%), highlighting the role of wastewater from laundry processes as a significant pollution source. Beaches close to unauthorized wastewater discharges, such as Anfi del Mar (*n* = 3) and El Puertillo (*n* = 32), showed the highest contamination levels, with a frequency of occurrence (FO) of microplastic particles of 67% and 58%, respectively. Playa de Las Nieves was the one with the lowest contamination level (*n* = 22), with a frequency of occurrence of microplastic particles of 41%. This is the first study to document microplastic ingestion in *C. ruber*, raising concerns about its ecological presence and the potential bioaccumulation of contaminants in marine ecosystems. Further research is essential to understand the long-term consequences of microplastic exposure on invasive species and their possible roles in pollutant transfer through food webs.

## Simple Summary

This study focuses on microplastic pollution in *Cronius ruber*, an invasive crab species found in the Canary Islands, and its connection to wastewater discharges. Researchers examined 63 crabs from four beaches around Gran Canaria and found that over half had microplastics in their stomachs. Most of these microplastics were fibers commonly used in textiles, revealing that wastewater—especially from laundry processes—plays a significant role in pollution. Beaches near unauthorized wastewater discharges showed higher levels of contamination, with Anfi del Mar and El Puertillo being the most affected. This is the first study to document microplastic ingestion in *C. ruber*, raising concerns about the ecological impacts and potential bioaccumulation of these pollutants.

## 1. Introduction

In recent decades, plastics have become one of the major ecological problems worldwide. Their massive production, combined with their low biodegradability and persistence in the environment, has steadily increased their accumulation in marine and terrestrial ecosystems. This phenomenon not only poses a challenge for their extraction, but also generates a growing problem due to the continuous diversification of their forms and contamination pathways.

In marine ecosystems, plastics have proven to be disruptive agents with ecologically and economically adverse effects. For example, abandoned or lost fishing nets contribute to the phenomenon known as ghost fishing, in which marine species are inadvertently trapped, compromising their survival and that of other associated species [1]. Furthermore, microplastics, defined as plastic fragments of less than 5 mm in diameter, represent a less visible but equally dangerous form of pollution, as they contain chemical additives that can act as carriers of toxic substances [2], causing problems in reproduction, development, and genetic integrity, even in organisms such as fish and crustaceans [3,4]. These compounds accumulate in the organisms that ingest them, generating bioaccumulative and biomagnifying effects in trophic chains [5].

An important yet frequently underestimated source of microplastics is the release of synthetic fibers from washing machines. A significant proportion of modern garments are composed of plastic-based materials, such as nylon, polyester, and acrylic, whose fibers are released during washing cycles. Domestic laundering contributes to microplastic pollution through the release of synthetic fibers. Factors such as the fabric type and washing conditions significantly influence the extent of fiber shedding from garments [6]. Furthermore, production techniques, material selection, and post-consumer care practices collectively determine a fabric’s propensity to shed microfibers [7]. Aggregated across millions of households, conventional laundry practices represent a major pathway for microplastic fibers to enter aquatic and terrestrial environments [8]. These fibers are assumed to accumulate along coastal areas near discharge points, potentially affecting the organisms inhabiting these areas.

**Figure 1:**
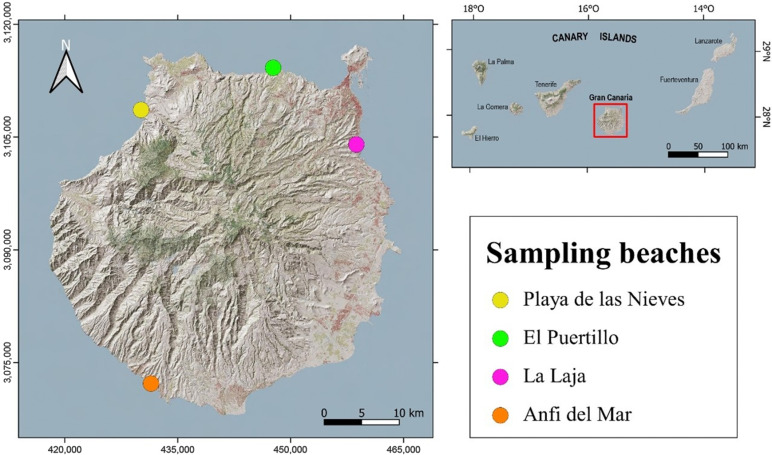
The location map of the sampling beaches. The map of Gran Canaria is in UTM coordinates (zone 28N, EPSG:32628): Playa de Las Nieves (28° 06′ 04,345″ N 15° 42′ 40,886″ W); La Laja Beach (28° 3′ 39,238″ N 15° 25′ 11,95″ W); El Puertillo Beach (28° 9′ 9,338″ N 15° 31′ 58,642″ W); Anfi del Mar Beach (27° 46′ 22″ N 15° 41′ 45″ W).

This study was carried out in Gran Canaria, belonging to the Canary Islands, an archipelago located in the North Atlantic Ocean off the west coast of Africa (Figure 1). These islands lie in the path of the Canary Current, a branch of the Azores Current, which transports pollutants from northern areas to their coasts.

Previous studies have demonstrated the accumulation of plastic pollutants on various beaches of the Canary Islands, including Gran Canaria [9,10,11,12], and their impacts on the marine biota, including microplastic ingestion by fish [13,14], sea birds [15], and jellyfish [16]. In this context, research on species such as the Atlantic m4ackerel (*Scomber colias*) has shown that 78% of individuals caught near the coast contained microplastics in their digestive tracts [14]. These findings underscore the need to assess the level of this contamination and its potential ecological and toxicological effects on other local species.

Previous studies have shown the presence of microplastics in crab species such as *Carcinus aestuarii* [17] or *Lithodes santolla* [18]. Some of these studies reveal the leaching of the microplastic particles into different tissues of the animal, such as the gills [19], digestive tract [20], hepatopancreas [21], or tissues [22]. Additionally, there is evidence of the impacts of these plastics on reproduction and embryonic development in individuals [23].

For this study, the species of interest is *Cronius ruber*, a predatory crab that has been reported as an invasive species in the Canary Islands since its first observation in 2016. It is speculated that the introduction of this crab to the islands may have occurred through maritime traffic or the movement of oil platforms within the archipelago [24]. Since then, its population has experienced exponential growth, and it is commonly found along the archipelago’s coasts. This crab occupies a significant ecological role as a generalist predator, feeding on annelids, bivalves, small fish, and other crustaceans, and competes with native crabs [25].

Our starting hypothesis is that *C. ruber*, inhabiting coastal waters near discharge points and feeding on benthic organisms and invertebrates, could be ingesting microplastics either directly or through its prey. This study aims to evaluate the presence of microplastics in this species, providing relevant data on the interactions between plastic pollution and invasive species such as *C. ruber*, as well as the potential impact of microplastics on the local trophic networks and marine ecosystems in the Canary Archipelago.

## 2. Methodology

### 2.1. Study Area

The Canary Islands are an archipelago located in the North Atlantic Ocean, off the west coast of Africa, between the coordinates (27°37′ and 29°25′ north latitude and 13°20′ and 18°10′ west latitude). These islands have a subtropical climate and temperate waters.

Crabs were collected from 4 beaches on the island of Gran Canaria (Figure 1): Playa de Las Nieves in the northwest, La Laja Beach in the northeast, El Puertillo Beach in the north, and Anfi del Mar Beach in the south. These beaches were chosen because of their proximity to illegal wastewater discharge points nearby, their ease of access, and the presence of the crabs.

### 2.2. Crab Harvesting

According to the methodology established by Triay-Portella [26], divers collected the crabs by hand using artificial lights during the night, as this is when *C. ruber* has its peak activity. Sampling was carried out at depths of 1 to 7 m on sandy and rocky bottoms during May, June, July, and October 2021. The maximum number of samples was sought at the four sampling locations, where a total of 63 samples were frozen (−20 °C) instantly after collection to maintain their quality.

### 2.3. Laboratory Procedures

The crab samples were kept in the freezer until dissection. Their stomach contents were placed in 10% KOH in glass beakers and completely covered with an alkaline solution. They were kept under digestion at 60 °C for a minimum of 24 h and a maximum of 72 h for samples that were not fully digested (Table 1). Prior to digestion, the stomachs were opened due to their hardness to facilitate the digestion of the organic material.

After the alkaline digestion was completed, the obtained samples were filtered through 25 µm metal filters using a suction pump.

Protective gloves and lab coats were worn throughout the sample analysis process, which was carried out inside a fume hood. All materials were thoroughly washed and inspected before use to avoid any possible contamination during the laboratory procedures.

Once the samples were filtered and dried, a visual inspection was carried out using a binocular stereomicroscope to identify any particles suspected to be microplastics. All suspected particles were photographed, measured (the particles averaged 0.7 ± 0.5 mm in length), and classified by type (fibers, films, and fragments) and color. To keep track of possible contamination during the latter process, a 25 μm mesh Petri dish was placed next to the microscope to control possible contamination in the air inside the laboratory.

### 2.4. Micro-FTIR Analysis

For the micro-FTIR analysis, the particles were analyzed using micro-Fourier transform infrared spectroscopy (micro-FTIR), using a Perkin-Elmer Spotlight 200i micro-FTIR instrument equipped with an MCT (Mercury Cadmium Telluride) detector. The instrument was operated in transmission mode on KBr disks with a spectral resolution of 8 cm^−1^ and a wavelength range of 550–4000 cm^−1^ [27] at the Interdepartmental Research Service (SIDI) of the Universidad Autónoma de Madrid. Beforehand, the samples were concentrated and refiltered. In this way, all suspected plastics were assembled and reorganized to obtain six glass plates with 57 particles to analyze. From these 57 particles, 70% (40 items) were analyzed, and 82.5% (33 items) were confirmed as plastic polymers.

### 2.5. Wastewater Discharge Point Locations

**Figure 2:**
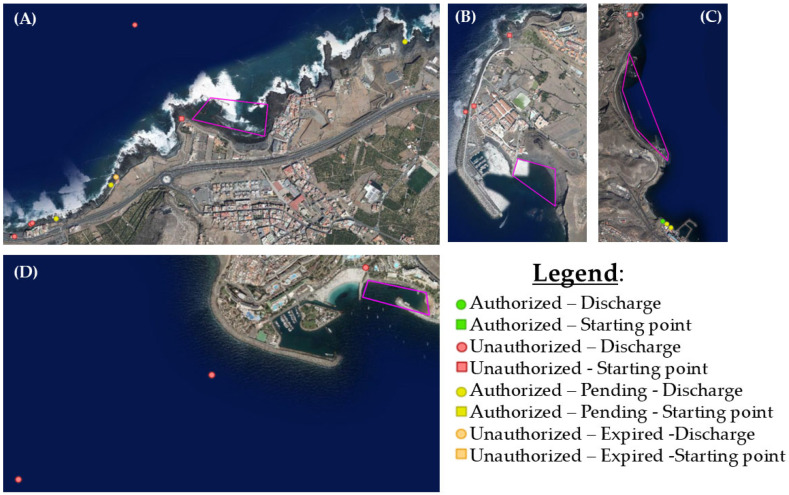
Images of the sampling locations represented by the purple area (200 m scale) with the nearby wastewater discharge points. The sampling area at each beach is outlined in pink. (**A**) El Puertillo Beach; (**B**) Playa de Las Nieves; (**C**) La Laja Beach; (**D**) Anfi del Mar Beach.

With the visor GRAFCAN from the IDE Canarias (Canary Government, https://visor.grafcan.es/visorweb/, accessed on April 2025), wastewater discharge points were tracked near the beaches where the crabs were collected. The discharges that were half a kilometer or less from the beaches sampled are shown in Figure 2.

### 2.6. Use of Artificial Intelligence

During the preparation of this manuscript, the authors used ChatGPT version GPT-4 for the purposes of rewriting to improve the language and readability. The authors have reviewed and edited the output and take full responsibility for the content of this publication.

## 3. Results

### 3.1. Frequency of Occurrence

Of the total number of crabs analyzed, 33 (52%) were suspected to be contaminated with microplastic particles in their stomachs. Fibers, films, and fragments were found.

In total, 57 suspect microplastic particles were identified across all samples analyzed. The average number of microplastic particles per contaminated individual was 1.73 ± 1.02 (mean ± SD), as shown in Table 2. Anfi del Mar Beach showed the highest contamination rate, with 67% of its samples contaminated (*n* = 3). In contrast, El Puertillo Beach showed a 58% contamination rate with the largest sample size (*n* = 32). Playa de Las Nieves had the lowest frequency of microplastic occurrence, with 41% in its 22 samples. At La Laja Beach, a frequency of 50% was detected in the six samples collected. Due to the highly unbalanced sample sizes between Anfi del Mar Beach (*n* = 3) and El Puertillo Beach (*n* = 32), the assumptions required for robust statistical analysis, including reliable estimates of variability and underlying distributional assumptions, were not met.

### 3.2. Characteristics of Microplastics

**Figure 3:**
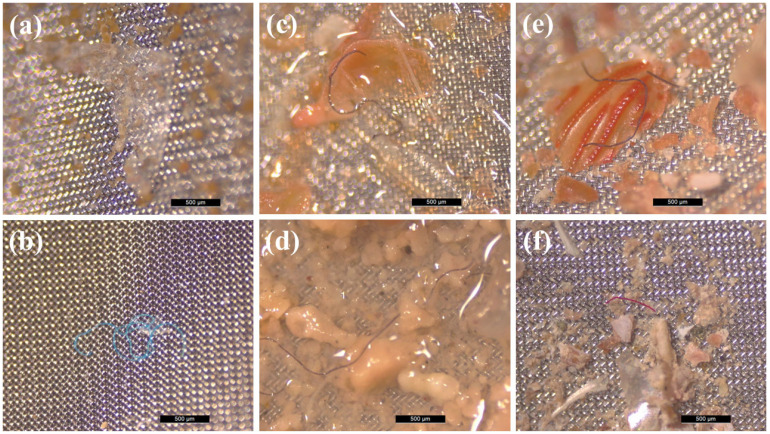
Photos of types and colors of microplastic particles which were found in stomachs of *Cronius ruber* specimens from Gran Canaria: (**a**) films (3.5%); (**b**) fragments (7%); (**c**) fibers (89.5%); (**d**) blue fiber; (**e**) black fiber; (**f**) red fiber.

Among the particles suspected of being microplastics, fibers, fragments, and films were found (Figure 3).

**Figure 4:**
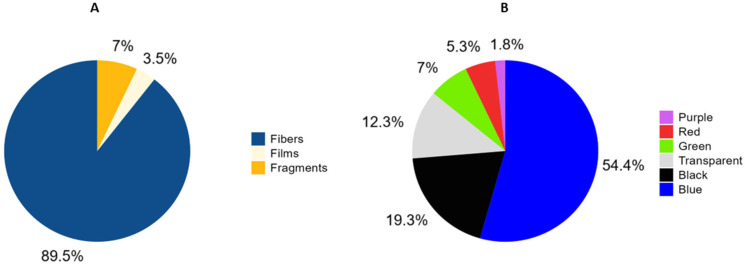
Distribution of suspected microplastic particle forms in percentages (**A**) and distribution of suspected microplastic colors in percentages (**B**).

As for the colors (Figure 4B), blue was the predominant color in most of the suspicious particles (55%). Black was the second most abundant color (19%), followed by transparent (12%). Green (7%), red (5%), and purple (2%) appeared in smaller proportions.

**Figure 5:**
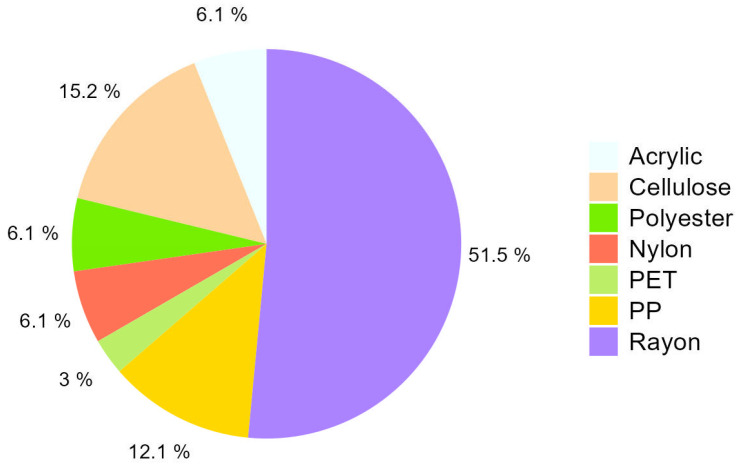
Types of polymer microplastics found in invasive *Cronius ruber* from Gran Canaria.

A total of 40 microplastics were analyzed, and the composition of 33 was confirmed by micro-FTIR (82.5%) (Figure 5). Rayon, a material from the textile industry, was the most frequent, with more than half of the microplastics identified as such. Other materials were cellulose (15.2%), polypropylene (PP, 12.1%), acrylic, nylon, and polyester (6.1% each), and polyethylene terephthalate (PET, 3.0%). The composition of all samples can be checked in the table attached as Table 3.

## 4. Discussion

More than half of the individuals examined had particles suspected of being microplastics in their stomach contents (52%). Of the 57 particles suspected to be microplastics, 40 particles were analyzed, of which the plastic composition of 33 (82.5%) was confirmed. Microplastic ingestion occurred at all sites (40–67% frequency), though the small samples limited the statistical comparisons.

As explained, the Canary Current brings with it plastic debris that is deposited on the islands of the Canary Archipelago, which acts as a natural barrier to the current. Although this pollution is quite high [9,10], the plastic pollution by fibers found on the beaches and in these four coastal areas could be explained by the presence of discharges close to them or by anthropogenic pressure [10].

In the case of Playa de Las Nieves, there is an unauthorized discharge close to the beach. At El Puertillo Beach, there are four unauthorized dumping points and four that are still being processed. Anfi del Mar Beach has three unauthorized dumping points nearby. Finally, La Laja Beach has one unauthorized discharge point on one side of the beach, while on the other side, there are several authorized and pending discharges a little further away. All of these wastewater discharges could expel microplastic (mainly fibers from laundry) pollutants that end up on the beaches, and therefore in the organisms themselves either directly through filtration, in the case of filter-feeding species, or through the simple confusion of this anthropogenic debris with the diets of certain generally small species. It can also occur indirectly by trophic transfer in the case of predatory species.

As specified in Figure 4A, 89% of the microplastic particles found corresponded to fibers, which was expected, since fibers are the most abundant type of pollution in the oceans [28]. According to the micro-FTIR analysis (Figure 5), a 52% content of rayon (a regenerated cellulose fiber that involves chemical modification with hazardous components [29]) was obtained. In addition, other types of materials used in clothing manufacturing, such as polyester, cellulose, or nylon, among others, were observed. Fiber pollution is closely related to wastewater discharges due to the use of washing machines, which are estimated to release more than 700,000 microplastic fibers in a single medium-load wash [6]. Fibers are readily ingested by filter feeders like bivalves, which are already known to filter these fibers and bioaccumulate [30,31,32].

The diet of *C. ruber* could also influence its ingestion of microplastics. As a generalist predator, this crab consumes a variety of prey, including annelids, fish, mollusks, and other crustaceans [25]. In the case of the mollusks, some of the species consumed are filter feeders. Several studies show how the presence of contaminants in prey affects predatory crab species [33,34,35,36], so *C. ruber* could suffer not only from bioaccumulation, but also biomagnification.

Of the colors found, the most abundant were blue, with 55%, and black, with 19% of occurrence, according to Figure 4B. Studies in other species show that these two colors tend to predominate in the stomach contents of marine organisms, together with white or transparent colors [37,38,39,40]. These higher concentrations of blue and black fibers could be explained by the wastewater discharges that carry fibers coming from washing machines, as explained above. These fibers would not have to be exclusively of plastic; they could be of other materials, but always of anthropogenic origin, being able to transport chemical products such as detergents or laundry conditioners [6,41]. Blue and black fibers are the most common fibers in clothing, and black fibers may also be discolored as they degrade into blue tones. It has also been observed that the ingestion of blue and black plastics occurs in fishes and other benthic crab species inhabiting coastal areas, so this possibility could be supported [42,43].

This is the first study that considers the possible existence of microplastics in the species *C. ruber*. There are no prior data or observations to compare with our results. In addition, the real impact that these contaminants could have in the long term on the digestive systems of the crabs is not yet known.

It is not yet possible to know how microplastics affect the species *C. ruber*, so future research should explore the ecological and physiological implications of microplastic contamination in *C. ruber*. Comparative studies across different geographical regions could provide valuable insights into the relationship between habitat-specific pollution and species-level responses. *Cronius ruber* appears to be a promising species for microplastic analysis, and could be further utilized in future studies on environmental contamination.

## 5. Conclusions

More than half (*n* = 33) of the *Cronius ruber* samples analyzed contained anthropogenic particles, of which 82.5% of the particles analyzed were microplastics and were detected at all locations. Fibers were the dominant type of microplastic found, accounting for 89% of the total, with rayon being the most common material, emphasizing the role of textile laundering as a major source of contamination. In all likelihood, the proximity to authorized and unauthorized wastewater discharge points contributed significantly to the contamination in the coastal areas.

The diet of *C. ruber*, which includes filter-feeding prey, suggests that the microplastics are ingested by trophic transfer. Blue and black fibers were the most abundant, supporting the textile origins and wastewater discharges, as these fibers are common in clothing and degrade over time in marine environments.

## Figures and Tables

**Table 1:** Summary of samples with basic information.

Location	ID	Sex	WSC (gr)	Year	Month	Day	Depth	Observation
Playa de Las Nieves	B017	1	1.5466	2021	6	9	2–5 m	24 h KOH
Playa de Las Nieves	B018	1	1.5635	2021	6	9	2–5 m	72 h KOH
Playa de Las Nieves	B019	1	0.727	2021	6	9	2–5 m	72 h KOH
Playa de Las Nieves	B020	1	0.3555	2021	6	9	2–5 m	72 h KOH
Playa de Las Nieves	B022	1	0.9287	2021	6	9	2–5 m	72 h KOH
Playa de Las Nieves	B023	1	0.3184	2021	6	9	2–5 m	24 h KOH
Playa de Las Nieves	B024	1	1.4	2021	6	9	2–5 m	24 h KOH
Playa de Las Nieves	B025	1	1.5889	2021	6	9	2–5 m	72 h KOH
Playa de Las Nieves	B026	1	0.86	2021	6	9	2–5 m	24 h KOH
Playa de Las Nieves	B027	2	1.7298	2021	6	9	2–5 m	72 h KOH
Playa de Las Nieves	B028	2	1.5815	2021	6	9	2–5 m	72 h KOH
Playa de Las Nieves	B029	2	0.8309	2021	6	9	2–5 m	24 h KOH
Playa de Las Nieves	B030	2	1.1597	2021	6	9	2–5 m	72 h KOH
Playa de Las Nieves	B031	2	0.3475	2021	6	9	2–5 m	24 h KOH
Playa de Las Nieves	B032	2	0.8549	2021	6	9	2–5 m	72 h KOH
Playa de Las Nieves	B033	2	2.3075	2021	6	9	2–5 m	24 h KOH
Playa de Las Nieves	B034	2	0.9357	2021	6	9	2–5 m	72 h KOH
Playa de Las Nieves	B035	2	0.844	2021	6	9	2–5 m	24 h KOH
Playa de Las Nieves	B036	2	0.796	2021	6	9	2–5 m	72 h KOH
Playa de Las Nieves	B037	2	0.52	2021	6	9	2–5 m	24 h KOH
Playa de Las Nieves	B038	2	1.364	2021	6	9	2–5 m	72 h KOH
Playa de Las Nieves	B039	2	1.44	2021	6	9	2–5 m	72 h KOH
La Laja Beach	B040	1	1.9705	2021	6	15	2–5 m	72 h KOH
La Laja Beach	B041	1	1.665	2021	6	15	2–5 m	24 h KOH
La Laja Beach	B042	1	1.0377	2021	6	15	2–5 m	72 h KOH
La Laja Beach	B043	1	0.6805	2021	6	15	2–5 m	72 h KOH
La Laja Beach	B044	2	0.5705	2021	6	15	2–5 m	24 h KOH
La Laja Beach	B045	2	0.3036	2021	6	15	2–5 m	72 h KOH
Anfi del Mar Beach	B046	2	1.723	2021	5	1	2–3 m	24 h KOH
Anfi del Mar Beach	B047	2	0.8472	2021	5	1	2–3 m	24 h KOH
Anfi del Mar Beach	B048	2	0.5424	2021	5	1	2–3 m	24 h KOH
El Puertillo Beach	B050	1	1.2897	2021	7	20	1–3 m	72 h KOH
El Puertillo Beach	B051	1	1.7483	2021	7	20	1–3 m	24 h KOH
El Puertillo Beach	B052	1	0.9359	2021	7	20	1–3 m	24 h KOH
El Puertillo Beach	B053	2	0.7529	2021	7	20	1–3 m	24 h KOH
El Puertillo Beach	B054	2	0.548	2021	7	20	1–3 m	72 h KOH
El Puertillo Beach	B056	1	0.6808	2021	7	27	1–3 m	24 h KOH
El Puertillo Beach	B057	1	0.6906	2021	7	27	1–3 m	24 h KOH
El Puertillo Beach	B058	1	1.7035	2021	7	27	1–3 m	24 h KOH
El Puertillo Beach	B059	1	1.265	2021	7	27	1–3 m	24 h KOH
El Puertillo Beach	B060	1	2.2029	2021	7	27	1–3 m	24 h KOH
El Puertillo Beach	B061	1	0.6934	2021	7	27	1–3 m	24 h KOH
El Puertillo Beach	B062	1	0.8472	2021	7	27	1–3 m	24 h KOH
El Puertillo Beach	B063	1	1.2546	2021	7	27	1–3 m	24 h KOH
El Puertillo Beach	B064	1	0.4226	2021	7	27	1–3 m	24 h KOH
El Puertillo Beach	B065	1	1.7793	2021	7	27	1–3 m	24 h KOH
El Puertillo Beach	B071	2	1.6161	2021	7	27	1–3 m	24 h KOH
El Puertillo Beach	B072	2	1.0271	2021	7	27	1–3 m	24 h KOH
El Puertillo Beach	B073	2	1.31	2021	7	27	1–3 m	24 h KOH
El Puertillo Beach	B074	2	0.5028	2021	7	27	1–3 m	24 h KOH
El Puertillo Beach	B075	2	1.5454	2021	7	27	1–3 m	24 h KOH
El Puertillo Beach	B076	2	1.4604	2021	7	27	1–3 m	24 h KOH
El Puertillo Beach	B077	2	0.7471	2021	7	27	1–3 m	72 h KOH
El Puertillo Beach	B078	2	1.0557	2021	7	27	1–3 m	24 h KOH
El Puertillo Beach	B079	2	0.5218	2021	7	27	1–3 m	24 h KOH
El Puertillo Beach	B080	2	0.756	2021	7	27	1–3 m	24 h KOH
El Puertillo Beach	B081	2	0.6707	2021	7	27	1–3 m	24 h KOH
El Puertillo Beach	B082	2	0.2323	2021	7	27	1–3 m	24 h KOH
El Puertillo Beach	B083	2	0.4822	2021	7	27	1–3 m	72 h KOH
El Puertillo Beach	B084	2	0.6083	2021	7	27	1–3 m	24 h KOH
El Puertillo Beach	B085	2	1.22	2021	10	1	1–4 m	24 h KOH
El Puertillo Beach	B086	2	1.2649	2021	10	1	1–4 m	24 h KOH
El Puertillo Beach	B087	1	-	2021	10	1	1–4 m	24 h KOH
El Puertillo Beach	B088	2	1.0579	2021	10	1	1–4 m	24 h KOH

ID = identification name. Sex: 1 = male, 2 = female. WSC (gr) = weight of the stomach contents of each crab in grams.

**Table 2:** Summary of data samples by location.

Location	*n*	Mean MP/ind	SD	FO%
Playa de Las Nieves	22	1.56	0.73	41
La Laja Beach	6	3.67	2.08	50
Anfi del Mar Beach	3	3.00	1.41	67
El Puertillo Beach	32	1.37	0.60	58
Total	63	1.73	1.10	52

*n*: number of samples; mean MP/ind: mean number of microplastics per individual; SD: standard deviation; FO%: frequency of occurrence of microplastic particles as percentage.

**Table 3:** Results of micro-FTIR analysis and their percentages of coincidence.

Sample	Type	Polymer	Coincidence (%)
1	Fiber	Cellulose	73
2	Fiber	NI	-
3	Fragment	NI	-
4	Fiber	NI	-
5	Fiber	Cellulose	87
6	Fiber	NI	-
7	Fiber	Polyethylene Terephthalate	83
8	Fiber	Rayon	85
9	Fiber	Polymethyl Methacrylate	83
10	Fiber	Rayon	67
11	Fiber	Cellulose	89
12	Fiber	Rayon	73
13	Fiber	NI	-
14	Fiber	Polypropylene	95
15	Fiber	Rayon	68
16	Fiber	Rayon	68
17	Fiber	Rayon	76
18	Fiber	Rayon	83
19	Fiber	Polyester	94
20	Fiber	Rayon	74
21	Fragment	NI	-
22	Fiber	Polypropylene	95
23	Fiber	Rayon	75
24	Fiber	Rayon	72
25	Fiber	Polyester	75
26	Fiber	Rayon	83
27	Fiber	PP	94
28	Fiber	Rayon	85
29	Fiber	Nylon	88
30	Fragment	Polymethyl Methacrylate	75
31	Fiber	Nylon	95
32	Fiber	Rayon	57
33	Fiber	Rayon	87
34	Fiber	Cellulose	70
35	Fiber	NI	-
36	Fiber	Rayon	71
37	Fiber	Rayon	69
38	Fiber	Rayon	79
39	Fiber	Polypropylene	91
40	Fiber	Cellulose	82

NI = No identification.

## Data Availability

The raw data supporting the conclusions of this article will be made available by the authors on request.
